# A salpingeal carcinoma revealed after prophylactic salpingoophorectomy in an asymptomatic BRCA1 carrier with breast malignancy

**DOI:** 10.1016/j.ijscr.2018.10.014

**Published:** 2018-10-12

**Authors:** Aris Giannos, Sofoklis Stavrou, Athanasios Douskos, Peter Drakakis, Dimitrios Loutradis

**Affiliations:** 1^st^OB.GYN Department, School of Medicine, National and Kapodistrian University of Athens, Alexandra Hospital, Lourou and Vasilissis Sofias Ave, 11528 Athens, Greece

**Keywords:** BRCA1-2 mutation, Bilateral mastectomy, Bilateral prophylactic salpingoophorectomy, Breast carcinoma, Salpingeal carcinoma

## Abstract

**Introduction:**

BRCA1 inherited mutation carriers face a lifetime risk of 72% to develop breast cancer and a percentage of 44% risk for ovarian cancer.

**Presentation of case:**

We present a case of a 35-year old Caucasian woman who, after the excision of a possible malignant finding on the right breast, was diagnosed with ductal breast carcinoma. The patient treatment included first chemotherapy and radiotherapy and then prophylactic bilateral mastectomy with plastic reconstructive surgery. At the age of 37, she underwent prophylactic laparoscopic bilateral salpingoophorectomy. The histological diagnosis of the surgical specimens was suggestive of a unilateral invasive high grade salpingeal cancer and the patient underwent an abdominal hysterectomy by laparotomy, omentectomy and bilateral pelvic lymphadenectomy.

**Discussion:**

Women with known BRCA mutations are strongly recommended to consider a risk reducing prophylactic salpingoophorectomy (RRSO) from their early 40 s to gain maximum benefit in cancer risk reduction from the procedure. The prevalence of occult carcinomas in BRCA1 carriers is almost 1, 5% if the prophylactic bilateral salpingoophorectomy is performed before the age of 40, while this percentage may be increased to 3, 8% for women who undergo the surgery between 40 and 49 years.

**Conclusion:**

We present an interesting case, of an accidentally revealed salpingeal carcinoma via RRSO in an asymptomatic young premenopausal woman.

## Highlights

• The median age between BRCA results receipt and RRSO should be reconsidered.
• Occasionally, no correlation exists between tumor size, malignancy or metastasis.
• Initial total excision of the mass, ensure the positive histological diagnosis.

## 1 Introduction

One to eight women have a lifetime risk to develop breast cancer in their life [1]. Somatic mutations in breast cells acquired during a person’s lifetime may be associated with most of breast cancer cases [1]. BRCA1 and BRCA2 inherited mutations have been well-described [1]; the BRCA1 gene composing of 22 exons, encodes a 220 kDa nuclear protein of 1863 amino acids [2]. Kuchenbaecker, in a recent study, showed that the BRCA1 risk of breast cancer was estimated at 72% and BRCA2 at 69%. In addition, ovarian malignancy risk was estimated at 44% for BRCA1 carriers and 17% for BRCA2 carriers [[3], [4], [5]].

Recent guidelines by experts recommend that women carriers of BRCA mutations should be offered risk reducing salpingoophorectomy (RRSO) by the age of 40 or after childbearing is completed [6]. The main purpose of this case report is to present an interesting case of a salpingeal carcinoma which was found accidentally via RRSO in a BRCA1 carrier with breast malignancy at the age of 37. This work has been reported according to the SCARE criteria [16].

## 2 Case presentation

A 35-year-old Greek female patient, gravida four and para two, presented to our breast unit department due to a non-palpable breast lesion which was revealed via ultrasonic examination, with malignant ultrasonographical features. Her personal medical history and her psychosocial history were uneventful and Pap Smear tests were up to date and all negative. She was non-smoker and consumed alcohol only in social occasions. Her body mass index (BMI) was 30, 48 Kg/m [2]. Because of her mother’s breast cancer history (diagnosed with breast cancer at the age of 50, but never tested for BRCA), the patient was followed up via transvaginal ultrasonography and breast ultrasonic examination every six months since 2004.

**Fig. 1:**
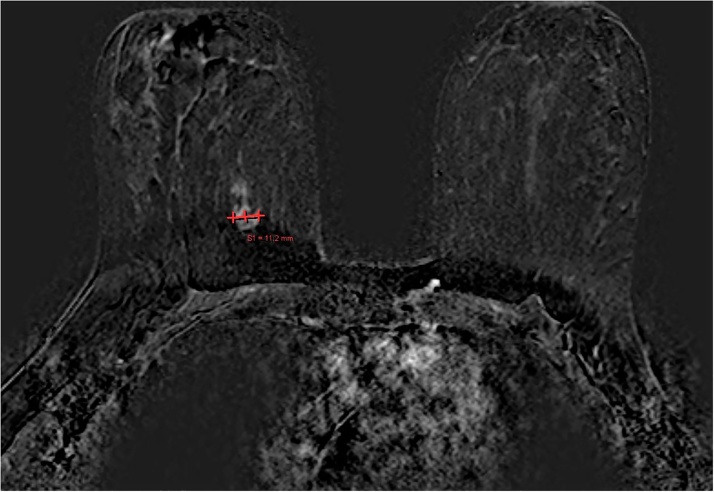
Lesion in the lower-inner quadrant of right breast with max diameter of 11.2 mm (MRM BIRADS IV).

Upon arrival, physical examination of her breasts did not reveal any palpable mass. Breast ultrasonography showed a hypoechoic lesion of 0,9 × 0,8 cm located in the lower inner quadrant, while breast magnetic resonance imaging confirmed the suspicious and possible malignant finding on her right breast (MRM BIRADS IV) (Fig. 1). The chest x-ray test was normal. Her laboratory workup was all normal.

**Fig. 2:**
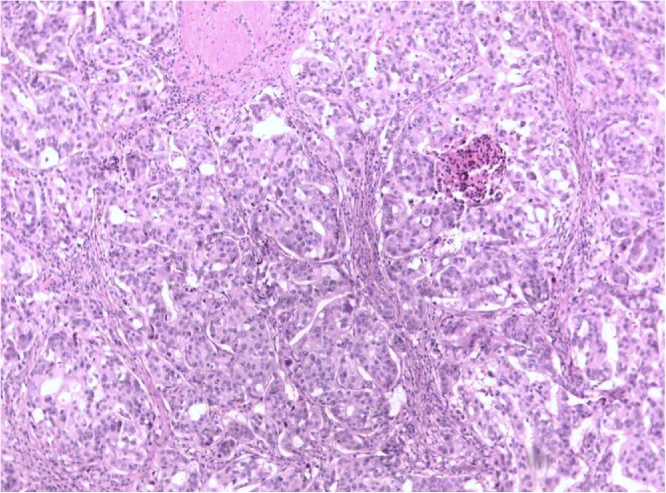
Breast cancer of no special type (NST- ductal) Grade II (Hematoxylin -Eosin X200).

Under general anesthesia, a total excision of the lesion (which had been marked via a hook wire) was performed by a gynecologist specialized in breast surgery with 6 years’ experience in breast surgical procedures. The normal tissue of the breast, the nipple and the areola were conserved. The excised mass was almost 1 cm in diameter. The contralateral breast was normal. Ultrasound guided biopsy was not performed because BIRADS IV was overestimated (not necessarily malignancy). There were no peri-operative complications and the breast healed well. Post-operatively, the patient was followed up in the breast ward. She was administered antibiotics, fluids and painkillers intravenously. Histology confirmed the ultrasonic diagnosis, revealing a central low grade invasive ductal carcinoma and a peripheral in situ ductal breast carcinoma grade III (Fig. 2). The margins of the resected surgical specimen were negative for cancer cells.

After almost a month from the first operation, the patient underwent a new surgical excision of unilateral right axillary lymphadenectomy of the first and second lymph nodes level. Twenty-two excised axillary lymph nodes were negative for metastasizing breast disease (0/22). Immunohistochemical analysis in cellular level showed ER (clone 6F11) stain positive in 100% of tumor cells, PR (clone 636) stain positive in 2% of tumor cells, Ki-67 (clone M1B1) stain positive in 60% of tumor cells and c-erb-2 (clone CB11)/HER 2 showing 3+ positive for cancer. Subsequently, the patient underwent treatment via chemotherapy, radiotherapy, herceptin and hormone therapy for the invasive ductal carcinoma Grade III (TNM staging: T1N0MO).

Gene evaluation for genetic mutations showed a BRCA 1 mutation; gene BRCA1 analysis was positive for mutations predisposing for breast or ovarian malignancy. More specifically the mutation p.Gly1738Arg (HGVS nomenclature)/ G1738R (BIC nomenclature) was detected. Thus, a prophylactic bilateral mastectomy was performed and followed by a successful plastic reconstructive surgery done by an experienced plastic surgeon, allowing for optimal aesthetic results.

**Fig. 3:**
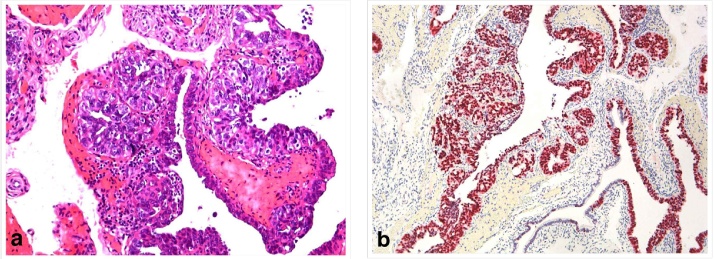
a) Fallopian tube with serous intraepithelial and minimally invasive carcinoma (Hematoxylin-Eosin X200). b) Fibrial serous carcinoma strong positive with immunohistochemical stain P53. Note the wild type expression in normal epithelium (X100).

A year after the first operation, the patient underwent a prophylactic laparoscopic bilateral salpingoophorectomy and uterine diagnostic curettage at the age of 37. Before surgery, blood tests including tumor markers were all in normal levels (CEA: 1,3 ng/ml, CA15-3: 6U/ml, CA125: 8U/ml, CA19-9: 30 U/ml, AFP: 1,2 IU/ml). The chest x-ray test was normal and the preoperative magnetic resonance imaging of the upper and lower abdomen did not detect any pathological finding. The histological diagnosis of the surgical specimens was suggestive of a unilateral invasive high grade salpingeal cancer, mostly intraepithelial and minimal invasive (0, 1 cm) serous carcinoma of one salpinx. Immunochemical analysis was positive for P53 and Ki67 (Fig. 3). No other pathologic finding was detected.

A further surgical evaluation was decided by the oncology council. Thus, the patient underwent an open total abdominal hysterectomy, omentectomy and bilateral pelvic lymphadenectomy by a gynecologist specialized in gynecologic oncology under general anesthesia. Postoperatively, the patient was followed up in a gynecology ward. She was administered antibiotics, fluids and painkillers intravenously. After a hospitalization of 5 days, she was discharged on the 6^th^ postoperative day, in good condition. She was prescribed tinzaparin (u) for 8 days and cefuroxime (u) peros for 7 days. The patient was compliant with the therapeutic program which was well tolerated with no significant side effects. Histology was totally normal without any other pathological findings. Bone scanning was normal and did not reveal areas of increased radionucleide uptake. Similarly, the hip and pelvis x-ray were normal.

The patient is in excellent clinical condition and she is followed up via ultrasonography of upper and lower abdomen and breast magnetic resonance imaging every 6 months, without any pathological finding after the last surgery.

## 3 Discussion

Women carriers of BRCA1 or BRCA2 mutation seem to have a potential risk of 72% and 69% to develop breast cancer and a risk of 44% and 17% to develop ovarian cancer respectively. Bilateral prophylactic salpingoophorectomy (BSO) is counseled as a prophylactic method because it has been shown to reduce the risk of ovarian cancer by 85–96% and of breast carcinoma risk by almost 50% in BRCA mutation carriers [9].

Women with known BRCA1/2 mutations are strongly recommended to consider a RRSO from their early 40 s in order to reduce their increased risk of fallopian tube, ovarian and breast carcinoma [7,10]. Via bilateral salpingoophorectomy, the surgeons remove both the two salpinx and the ovaries bilaterally. The theory of tubal origin of ovarian cancer is well-known. According to this, ovarian malignancy may arise from a precursor malignant lesion in the fallopian tube (tubal intraepithelial carcinoma), which leads to an invasive, high-grade, type II carcinoma ultimately involving the ovary itself [11,12].

Risk-reducing salpingectomy (RRS) upon completion of childbearing, with delayed oophorectomy (RRO) beyond the currently recommended age, offers an early, potentially risk-reducing intervention, with the advantage of postponement of premature menopause and its effect on non cancer-related morbidity and (menopause-related) quality of life [17,18].

Amy Finch et al in 2014, showed that the prevalence of occult carcinomas for BRCA1 carriers was almost 1,5%, if the surgery has been performed before the age of 40, while this percentage may be increased to 3,8% for women who undergo prophylactic bilateral salpingoophorectomy between 40 and 49 years. These data supported the recommendation for BRCA1 carriers to undergo the above surgical procedure at the age of 35 or once childbearing is completed [8]. Additionally, the woman – BRCA1 carrier who chooses to undergo bilateral salpingoophorectomy more closely to the age of 40, may face an increased risk of ovarian cancer which is estimated to be 4%. If the same patient chooses to delay the time of prophylactic surgery until the age of 50, the probability will increase to 14.2% [8].

Based on several studies, the majority of BRCA carriers undergo BSO shortly after the results of genetic tests [[13], [14], [15]]. Bradbury et al. in 2008, showed via a cohort study, that many women decided to have bilateral salpingoophorectomy more than 12 months after BRCA mutation was found; the median time was 12, 5 months, while some patients waited a lot of months to several years for the surgery [9].

In our case, we did not perform paraortic lymph node excision (LNE), because of the small size of the tumor, the low grade and the fact that we had no imaging findings. We informed the patient about the choice of sentinel- LNE, but her decision was to go for a total excision of the mass (she felt more secure). Finally we did not perform LNE during the first surgery, because we excised the mass until tumor free margins and previous imaging showed benign features. In addition, a valid histological result is attainable for tumors over 1 cm.

## 4 Conclusions

In our case, the patient was diagnosed with salpingeal carcinoma within 2 years after the finding of BRCA gene mutations at the age of 37. Although many similar cases have been described, the uniqueness about our case is the fact that a young woman with an insignificant tumor mass gave metastases to the salpinx shortly after the initial diagnosis. The current standard RRSO at age 35–40(BRCA1) or 40–45 (BRCA2) is highly effective in reducing ovarian cancer incidence. However, consequent premature surgical menopause comes with short- and long-term non cancer-related morbidity and probably affects quality of life.

## Conflicts of interest

NA.

## Funding source

NA.

## Ethical approval

IRB/Ethics Committee ruled that approval was not required for this study.

## Consent

Written informed consent was obtained from the patient, and this has been stated in the manuscript per the submission instructions.

## Author contribution

Conception and design of study: Athanasios Douskos, Sofoklis Stavrou.

Acquisition of data: Athanasios Douskos, Sofoklis Stavrou, Aris Giannos.

Analysis and/or interpretation of data: Athanasios Douskos, Sofoklis Stavrou, Aris Giannos.

Drafting the manuscript: Athanasios Douskos, Sofoklis Stavrou, Aris Giannos.

Revising the manuscript critically for important intellectual content: Peter Drakakis, Dimitrios Loutradis.

Approval of the version of the manuscript to be published: Aris Giannos, Sofoklis Stavrou, Athanasios Douskos, Peter Drakakis, Dimitrios Loutradis.

## Registration of research studies

As this is not a ‘first-in-man’ case study, our paper is not eligible to be registered.

## Guarantor

Aris Giannos.

Sofoklis Stavrou.

Athanasios Douskos.

Peter Drakakis.

Dimitrios Loutradis.

## Provenance and peer review

Not commissioned, externally peer reviewed.
